# Service Marketing in Online Shopping Platform: Psychological and Behavioral Dimensions

**DOI:** 10.3389/fpsyg.2021.759445

**Published:** 2021-10-21

**Authors:** Yong Wang, Manci Qi, Liz Parsons, Fu-Sheng Tsai

**Affiliations:** ^1^Business School, Huaiyin Institute of Technology, Huai'an, China; ^2^Doctoral Program, I-Shou University, Taiwan, China; ^3^Management School, University of Liverpool, Liverpool, United Kingdom; ^4^North China University of Water Resources and Electric Power, Zhengzhou, China; ^5^Department of Business Administration, Cheng Shiu University, Kaohsiung, Taiwan; ^6^Center for Environmental Toxin and Emerging-Contaminant Research, Cheng Shiu University, Kaohsiung, Taiwan; ^7^Super Micro Mass Research and Technology Center, Cheng Shiu University, Kaohsiung, Taiwan

**Keywords:** service marketing, online shopping, platform, psychological antecedents, behavioral antecedents

## Abstract

This conceptual analysis critically discusses how service marketing is workable for online shopping platforms and how important service-related and influenced factors played their roles the aforementioned issue. The concepts of service, service marketing, and related factors were re-visited, or at least reflected, in the new context of online platforms. Mostly, we framed the essence and importance of those discussed factors from the psychological and behavioral angles. Implications for theory, practices, and policy-making were offered seriously.

## Introduction

Recent years have witnessed the huge interests in the trending research on service marketing (Rust and Huang, 2014). The researchers have highlighted that service is one of the most important section of marketing that could be felt by consumers before the consumption, and thus could influence the purchase decision directly (Rust and Huang, 2014). With the development of service marketing, this concept has been extended from physical to virtual marketplace in various forms (Schultz et al., 2013). When people reach nearly all the shopping platforms, the concept of “service” goes beyond the traditional understanding and increasingly covers other new conceptual elements (Shareef et al., 2016; Al-kumaim et al., 2021).

Nevertheless, with the development of online shopping, it is increasingly difficult to evaluate which high-quality standards online shopping platforms should have. Such unclear standards caused interest of researcher, to systematically examine how service marketing could be applied in the online shopping platforms to contribute to platform performance. Doing so, a conceptual analysis provided by this paper can contribute in three ways. First, we could explicate the erratic and fuzzy standards to judge the effectiveness of service in online platform contexts, which help improve the comprehensiveness and conceptualization for further (qualitative and quantitative) studies. Second, for practitioners of online shopping, the review and analytical arguments of this study could provide theoretical support about how to develop the quality service mechanisms (procedures, rules, references, etc.). Third, for policymakers, a clearer description about the standards of service marketing in online shopping platforms can facilitate reasonable policies formation to deal with both the civil and commercial disputes related to the services of online shopping platforms.

Service marketing has become one of main subfields of marketing. The value of service marketing lies in the expectations and reactions of consumers (Fan and Dong, 2021). The services may be offered directly or indirectly to the consumers in business to consumer (B2C) or business to businesses (B2B) (Rust and Huang, 2014). With increasing popularity, the online shopping platforms have shown their potentials to replace the traditional shopping mechanisms. Generally, the traditional marketing mix is also working in the online shopping platforms, for which the consumers are still influenced by 4Ps (Product, Price, Place, and Promotion) even if the effects of “place” has been weakened to some extents (Rust and Huang, 2014). Namely, the traditional 4Ps are still able to be extended by service marketing in the online platforms. However, as an invisible form of marketing, it is more difficult to be managed when it is applied on a virtual network platform. Customer service is crucial for online shopping platforms that demands high level of customer loyalty based on customer perceptions of the platform's service quality.

Unfortunately, there is still a lack of commonly accepted answer about what standards could be used for online platforms. Due to such vagueness, various online shopping platforms are unable to create their own high-quality service marketing clearly and effectively. Even if their service quality is improving, with deep understanding of service, the cost of these attempts is not only capital investment but also the patience and evaluation of the consumers (Chang et al., 2016).

Against such background, this article tends to reference related literature to conduct an integrative conceptual analysis for the issues mentioned above.

## The Conceptual Analysis

Most of the extant studies direct to a common goal of explaining why online shopping platforms should improve service. In the earlier research, the importance of service and service marketing did not receive enough attention, for some studies even believed that the online shopping did not incorporate service concepts. However, with development of service, this claim has been overturned by further studies who claimed that the service quality could increase the retention rate of consumers. In addition, the studies form Hsieh and Tsao (2014) also stressed that the high-quality service of online shopping platform would reduce the perceived risk of consumers and further increase the consumer loyalty.

After that, the researcher discussed the factors which could influence the service marketing in online shopping platforms to find out the method to improve service quality. Afterward, claimed customer commitment would also influence the consumer perception of services of online shopping (Singh and Pandy, 2016), which has been proved by other studies. Finally, for consumers, the perceived services would also be different according to the different values (Blasco-Arcas et al., 2014; Chang et al., 2016).

Finally, the researcher collected related opinions about how online shopping platforms could improve the service quality. In some studies, it was mentioned that the consumer satisfaction of service quality is highly depended on the ease of use of website pages rather than the personalization. Moreover, with the development of online shopping portals, the uses of shopping platforms are not only limited for purchasing but also used as communication channels at present. Therefore, Lazarus et al. (2014) put forward that online shopping platforms, especially for some platforms that highly rely on the mobile channels, some factors should be given priority to the consideration, such as convenience, security, and emotional values. The last but not the least, Chang et al. (2016) and Zheng et al. (2017) found out that the coupon proneness and value consciousness play important roles in explaining the e-loyalty.

### Service Marketing

In the existing studies, the importance of service marketing has been accepted widely by scholars and practitioners.

With the forming of unique concept of service, service marketing has become an important subject of marketing. According to Haynes and Grugulis (2014), the service is believed to include four main characteristics, such as involving intangibility, inseparability, perishability, and variability, which is a commonly accepted opinion. First, service is a concept that lacks physical form, which means that service does not interact with consumers through any conventional senses. The value of service is created by the consumption or experience, as its ownership could not be transferred, so that the quality of service is unable to be evaluated before purchasing. Inseparability simply means that the production and consumption are inseparable, so that the service marketing is also influenced by this characteristic that the process of service marketing is highly contacted and labor-intensive. Through the impact of inseparability, the companies that focus on the service marketing are easier to be influenced by the capital for labor and human error. Moreover, service is ephemeral and unable to be stored. In other words, the supply of service could not have buffer between the supply and demand, because all supply should be provided timely. The last characteristic of service is the variability, also known as heterogeneity, which states that the services are inherently variable in quality and substance. Namely, service quality is difficult to manage and there are fewer opportunities to standardize the service marketing delivery. According to the abovementioned characteristics, the unique characteristics of services give rise to the problems and challenges of service marketing that are rarely paralleled in product marketing. These challenges and problems would be further discussed as follow.

### The Classification of Service

In current theories, the framework of service marketing has been controversial according to various standards to distinguish different types of services. The first classification is related to who or what is being processed, involving people processing, mental stimulus processing, possession processing, and information processing (Lazarus et al., 2014). This type of classification mainly relates to the sources of core value generated with service. In addition, there is another method to classify service marketing according to the degree of customer interaction, involving high low contact services (Yahyaoui et al., 2015).

However, the quality of services is difficult to judge before purchasing (Yahyaoui et al., 2015), so that economists believe that nearly all of service marketing could be classified with a processual framework of “Search → Experience → Credence (SEC)” (Girard and Dion, 2010). According to this framework, most products fall into the search goods category, those which possess attributes that can be evaluated prior to purchase or consumption. Experience goods mean products or services that can be accurately evaluated only after the product has been purchased and experienced, which could be seen as another side of search products. It is different to the above two classifications, credence claims are the goods that are difficult or impossible to evaluate even after consumption has occurred (Girard and Dion, 2010). These goods are called credence products because the quality evaluations of the consumers depend entirely on the trust given to the product manufacturer or service provider (Girard and Dion, 2010). According to these different classifications, the forms of service marketing operated in online shopping platforms are also different, so that the content of this section would be used in further discussion below.

### Service and Service Marketing in Online Shopping Platforms

Due to the virtuality of online shopping platform, the importance of service and service marketing did not pay enough attention, to which some earlier research even believed that the online shopping did not have the concept of service. Service goes with different face in physical vs. online world. In a previous study, the researchers claimed that the online shopping is a new concept that could subvert over the position of traditional shopping and get rid of the limitation of services provided by humans. Some researchers also put forward a similar opinion that the online shopping based on machinery and procedures did not have the ability to exchange emotion with consumers, so that consumers would not have demands and expectation emotionally, which could create a fair competitive channel because the consumer would only focus on the quality of products (see also Wei, 2021). This claim seems reasonable according to the background of era, but it has been denied at present. In the further analytical study, they found out the optimal service level on the “fulfillment and responsiveness” function for the risk averse uniquely exists. Customer loyalty is more positively correlated to the service level, which could cause the largest optimal service level. Moreover, the business organizations do not have to worry about the increase of cost on service-construction would reduce profits, because it was found out that the optimal service level is independent of the profit target, which states that the profits of organizations would not be reduced by the improvement of service. In other words, if the organization could achieve nearly optimal service level, no matter how organizations create its targets, the service quality would only increase the retention rate of consumers.

Based on the above discussion, in nature, physical vs. online service marketing differ in the following ways. First, the medium of service marketing delivery is different (e.g., Majeed et al., 2020). While service marketing delivery can be done through touchable medium, which can be stored, transferred, and removed in physical ways, and online service marketing can only be passed *via* a virtual medium, which has higher level of dangers to be easily copied or removed. The different medium of service marketing delivery also affects the ways the stakeholders interact *via* the marketing medium (Méndez-Aparicio et al., 2017). Also, the customers experience differently (Wong et al., 2018). Second, the physical and online service marketing differ in the scope and speed of marketing outcomes. The range of influences can be broader by online service marketing, and for online service marketing the marketers could see the results of their strategies faster. Third, also due to the aforementioned difference in scope and speed, the online service marketing relatively relies on the technological advantages, such as artificial intelligence and big data, which offer more instant feedback for service marketing modification during the marketing contacts. The need for precision is higher in online service marketing as compared with the physical service marketing.

### The Perceived Risk Determines Online Loyalty

As mentioned above, the service quality is more related to the consumer perception than profit target, but it also means that the service of online shopping highly relates to every factor that could be perceived by the consumers, such as risks. The study of Hsieh and Tsao (2014) is highly representative among all the studies discussed about perceived risks, in which they analyzed the role of perceived risk within the services of online shopping platform in-depth. To discuss the reason why it is necessary to mention perceived risk, Lazarus et al. (2014) provided a statement that perceived risk has a significant negative effect on online loyalty, which states low perceived risk means higher online consumer loyalty. In the study of Hsieh and Tsao (2014), they mentioned that system quality and information quality do not have significant negative effects on the perceived risk, which seems an opposite opinion with Lazarus et al. (2014). However, Lazarus et al. (2014) only claimed that the security should have a priority to consideration, but they did not mention it would influence the perceived risk. Therefore, the study of Hsieh and Tsao (2014) is a supplementary explanation of Lazarus et al. (2014). Moreover, Hsieh and Tsao (2014) found out that e-service quality has a significant negative effect on perceived risk, which means high-quality service of online shopping platform would reduce the perceived risk of consumers. Managerially, this paper offered a conceptual foundation for service marketing tactics for online platform.

### The Consumer Loyalty Equal to Success

It is clear that the studies of Hsieh and Tsao (2014) mentioned that the service is related to the consumer loyalty, but they did not further explain how important consumer loyalty is in the operation of organizations. In fact, it has been mentioned by other researchers that the importance of consumer loyalty directly influenced the success of companies in long-term. The consumer loyalty, or called as customer retention, plays an irreplaceable role in the operation of online shopping platforms, but the situation is that only a few of managers could really understand its significances. First of all, the direct influence of consumer loyalty is to increase the repurchase intention that the high evaluation of online services or high consumer loyalty could largely increase the possibility of repurchasing. According to the results of model based on the Information Systems (IS) use theory and social exchange theory, it was found that the shopping habit increases the influence of emotional evaluation on continuance, while habit weakens the impact of rational evaluation on continuance intention. Some researchers similarly conclude that trust and perceived benefits are the key predictors of the consumer attitudes toward online shopping, as 28% of the variation in online shopping attitudes was caused by the perceived benefits and trust. In other words, a higher brand loyalty or repurchase rate could reduce the impacts of rational evaluation, and further increase fault tolerance in competition and the rate of success.

Therefore, according to some research, the service quality will increase the retention rate of consumers directly, which is independent of the profit target. In addition to the high-quality services, the perceived risk of consumers played the important role when organizations intend to increase consumer loyalty, and consumer loyalty directly influenced the success of companies in long-term.

### The Factors to Determine the Quality of Service in the Online Shopping Platforms

#### Personal Privacy

According to the existing studies, most of the researchers believe safety to be one of the most important factors that would influence the consumer perception of services. As a virtual trading platform, online shopping platforms are more prone to the security issues, which directly influence the service quality of platforms. In a previous study, it was mentioned that perceived Web security and personal privacy concerns can influence the consumer acceptance of online shopping. This opinion was accepted by some researchers in their study which claimed that the users form perceptions of security control that strongly determine how much trust they put in online services. However, security control is a difficult measure to observe the credence quality of online services that Internet users cannot easily assess through the research or experience. Moreover, in addition to qualitative analyses, the importance of safety is also supported by some quantitative data. For example, a survey was created and 120 questionnaires were distributed among the students and the general public. The results showed that people already shopping online prefer to purchase online in future, because of the most important privacy factors. Therefore, according to the current studies, the personal privacy is one of most influential factors to influence the consumer perception of service marketing in online platforms.

#### Commitment for Customers

In addition to safety, some studies claimed customer commitment would influence the consumer perception of services of online shopping. It was found out that different attitudes of consumers toward online shopping shows that they would still prefer the traditional shopping pattern because of various traits related to promises, such as value, trust, and comfortable. At the same time, the key antecedents and consequences of marketing in online retailing highly rely on four mediators, involving trust, commitment, relationship quality, and relationship satisfaction. A novel multiple-criteria decision-making (MCDM) approach is used to solve the decision of service quality for shopping platform services. They also claimed that similarity and seller expertise were found to have the strongest impact on the relational mediators. Indeed, the security control perceptions of the users arise solely from their predispositions, but online service providers can influence the consumers. Thus, existing studies concluded that the commitment of supplier in online platforms would highly influence the attitude of consumers because word of mouth was the most critical outcome of relationship marketing efforts.

#### Values of Consumers

However, it was showed that the determinants of online shopping acceptance differ among the service types, but the previous studies have limited the generalizability of their results to a few products at best. The “fulfillment and responsiveness” function is significantly related to the customer loyalty in online shopping platforms. However, they still identified a research gap about whether the customer loyalty would be influenced by the individual values. In the research of Chang et al. (2016), the researchers intended to discuss this research gap, and researchers used 866 samples to explore the relationships among the intrinsic motivation, extrinsic motivation, flow, cognitive attitudes, perceived satisfaction, and purchase intention of online shopping of the consumers from a cognitive attitude perspective. The results indicated that hedonic value, utilitarian value, security, and privacy significantly affected the cognitive attitudes (i.e., cognitive trust and perceived risk). The results of Shu-Hao Chang et al. (2016) initially proved that the individual values of consumers would influence the effects of consumer loyalty, which the perceived services would also be different according to different values. Blasco-Arcas et al. (2014) claimed that the online cues related to customer to customer (C2C) interactions and coproduction in the engagement platform determine the customer co-creation experiences (Razmus, 2021; Siddique et al., 2021). For example, if the customers perceive that they are co-creating the experience, their purchase intentions increase (Razmus, 2021; Siddique et al., 2021).

Thus, for the discussion about what factors would affect the service in online shopping platforms, some most important factors have been found out by existing study, such as personal privacy, commitment for customers, and values of consumers. Compared with the other factors, the personal privacy is the most influential factors to influence the consumer perception of service marketing in online platforms. In addition, the role of commitment of supplier in online platforms is that it highly influences the attitude of consumers, because word of mouth is the most critical outcome of relationship marketing efforts. Finally, even if the values of consumers are less influenced by online platforms, individual values are still able to influence the effects of consumer loyalty, due to different perceived standards.

## Online Platform Service Quality Improvement

### The Ease of Use

As discussed before, there were three main factors that would highly influence the consumer perception of service in online shopping platforms. Thus, in this section, researcher intends to analyse how service marketing could be applied in online shopping platforms. In fact, this is not a new topic in academia; a research model was developed to examine the relationship among the service quality dimensions and overall service quality, customer satisfaction, and purchase intentions. The result seems outdated at present due to fast development of online shopping in past 10 years, but it still explained how early scholars understood service marketing of online shopping platforms. In this study, the researcher collected data from a survey of 297 online consumers to test the research model. The analytical results showed that the dimensions of website design, reliability, responsiveness, and trust affect overall service quality and customer satisfaction. Moreover, service quality is significantly related to the customer purchase intentions. However, the personalization dimension is not significantly related to overall service quality and customer satisfaction. In other words, in earlier period, the consumer satisfaction of service quality is highly depended on the ease of use of website pages rather than the personalization.

### The Security of Shopping

With the development of online shopping, the uses of shopping platforms are not only limited to the purchasing but also as communication channels at present. Therefore, the uses of online shopping platforms have been classified into social networking rather than pure functional websites. As a result, Lazarus et al. (2014) used to mentioned that the interaction-centric capabilities for engaging consumers are the basis of “co-creation capabilities” of a firm. These capabilities can be used as the strategic tools to develop competitive advantage for service firms under service-dominant logic. Past literature has indicated that the consumption value is an important factor in consumer decision-making on whether to adopt online shopping. However, the previous studies of the indirect effects of personal characteristics on the adoption of online shopping have emphasized solely the importance of utilitarian values, but none have investigated the indirect effects of consumption values that include both utilitarian and hedonic aspects. As a result, in this research, the researchers discussed how online shopping platforms could use the consumption values and personal characteristics to carry out high-quality services. The results showed that online shopping platforms, especially for some platforms that highly rely on mobile channels, some factors should be given priority to consideration, such as convenience, security, and emotional values.

### The Role of Promotion

As a type of shopping method, the promotion could play a positive role to increase the consumer satisfaction. Moreover, in the later research from Chang et al. (2016), 866 samples were collected and analyzed using the structural equation modeling for validation of the proposed model. They found out that the cognitive attitudes significantly affected the perceived satisfaction and purchase intention, respectively. Flow significantly and positively influenced the cognitive trust and purchase intentions, respectively. The cognitive trust is the mediators between motivations and perceived satisfaction/purchase intention. For how could improve the perceived satisfaction and purchase intention, in addition to the traditional or mentioned methods, Zheng et al. (2017) used a sample of 537 users of an online shopping platform to advance the theoretical understanding of e-loyalty by exploring the roles of coupon proneness and value consciousness in the context of online shopping platforms. They found out that coupon proneness and value consciousness play important roles in explaining the e-loyalty.

In other words, the quality of service operation on online platforms is highly depended on the ease of use of website pages rather than the personalization. However, with the development of technology, the demands of security are also increased in the past few years.

## Discussions

This research aimed to discuss the role of service marketing in online shopping platforms and in-depth discuss the importance of service marketing in the marketing of online shopping platforms. However, even if the concept of “service” has had a clear definition, it is still a visible product that its value only depends on the gap between the consumer expectation and real experience. Moreover, as one of subfields of marketing, service marketing and product marketing are always combined in the marketing strategies, so that the discussion of service marketing in online platforms cannot be fully independent of the influence of product marketing. Thus, another disadvantage of this type of studies is that the performance of service marketing is highly influenced by the products, as there are different products that are sold on online shopping platforms. In other words, invisibility of service marketing and dependency with product marketing caused the research aim difficult to be achieved.

In the current study, the importance of services for online shopping platforms has been discussed in-depth, in which the researchers not only analyzed how service influence the success of organizations but also what method could be used by these organizations. Service marketing, as one of main subfields of marketing, is more difficult to be managed when it is applied on a virtual network platform. However, with the development of service, this concept has been paid more attention than before at present, so business organizations need to put enough attention on the improvement of service quality.

### Theoretical Implications

First, as an academic literature review, this paper reviewed numerous representative studies in past 15 years, so that this paper could give other researcher a guide of future projects. In addition, service marketing is becoming increasingly popular in recent years, as most of the marketing scholars are interested in this field. Therefore, the topic area of this study is a hot topic that would not be outdated in a short time.

Even if this paper conceptually analyzed the extant literature systematically, there are still some possibilities for the future studies. These research gaps are divided into the guesses that have not been proved and the questions put forward by the current study. The first opportunity is related to the questions mentioned left by earlier studies. Previous studies mentioned that online shopping based on machinery and procedures did not have the ability to exchange emotion with consumers; however, it was found if an organization could achieve nearly optimal service level, the service quality would only increase the retention rate of consumers. However, the previous studies did not explain the optimal service level or provide the framework to test the optimal service level. Even if a researcher has found some factors that could influence the service quality, it is still unable to answer this question. Thus, the first opportunity is to know how online shopping platform could create the optimal service level or how these organizations divide into different levels.

Second, as early as 2005, Guang and Fen have found that the consumer satisfaction of service quality is highly dependent on the ease of use of website pages rather than the personalization. Even in 2015, Assarut and Eiamkanchanalai mentioned that online shopping platforms should provide priority to consider some factors, such as convenience. Both these studies did not discuss the role of personalization, but some research (e.g., Koch and Benlian, 2015; Zobov et al., 2016; Oberoi et al., 2017) claimed personalization has become increasingly important at present, so the second opportunity is to prove whether personalization has become more important than some factors, especially in the current era.

Third, the most important contribution of this study is that it provided experiences about long-term and voluminous literature review, laid the foundation of future research. Moreover, the topic of this project is about service marketing and online shopping platform, created a systemic review about the concepts about these fields in-depth.

Fourth, the current conceptual analysis discussed the psychological and behavioral aspects of the important issues separately. Future studies are encouraged to discuss, or empirically examine, the issues that are at the intersection of the two major perspectives. As individual psychology and behaviors could be mutually influential, more interesting phenomena and issues of service marketing in online shopping context might be explored by integrating the two perspectives.

Fifth, future research should take the role played by culture into account when investigating the service marketing in online shopping context. Here, culture not only refers to that in physical settings (societal, social, organizational, etc.) but also that embedded in virtual and neuro-psychological worlds. In the age of internet and more modernized technologies, the way culture forms and functions are very different from that in traditional business settings. Whether in physical or virtual settings, however, one thing stays for sure is that culture could affect the human cognition, interactions, decisions, and actions. But in the virtual world, culture becomes more difficult to capture and measure. So, it would be quiet challenging but contributively if scholars of online service marketing could bring the updated concept of culture into related studies.

Last, the performance of service marketing is highly influenced by the products as different products are sold on platforms. In other words, the invisibility of service marketing and dependency with product marketing caused the research aim difficult to be achieved.

### Practical Implications

For related practitioners of online shopping, the results of this study could provide theoretical support about how to develop quality of service and how to increase the effectiveness of service marketing. The literatures reviewed in this paper involved the influential factors, operating method of service marketing, impacts of service marketing on consumers, and the methods of improvement, which intended to help online platforms to find out a method to create high-quality service. However, even if the concept of “service” has had a clear definition, it is still a visible product that its value only depends on the gap between the consumer expectation and real perception. This paper explained the related concepts of service in details, which could help practitioners to further understand the service and its importance. Moreover, with the development of technology, the online shopping is able to replace more and more functions of real stores, to become mainstream shopping method in the future. In addition, the findings of this research could contribute in changing online shopping platform to be more humane and more advanced, which could even change the current business philosophy of these platforms to pay more attention on service marketing.

### Policy Implications

For policymakers, a clearer analysis of service marketing standards in online shopping platform context contributes to policy regulations for online shopping platforms' service-related disputes. Service marketing, as one of main subfields of marketing, is more difficult to be managed when it is applied on a virtual network platform. Thus, it is easier to have disputes between online retailers and consumers, due to service problems and lack of related laws to regulate the market. However, this paper provides a clear description about how consumers require the service quality of online shopping, so it helps the current market to establish the market standards.

### Concluding Remarks

With the development of service marketing, this concept has not been limited only on real places, which it has be extended to online communication and been reflected by various forms. However, with the development of online shopping, it is increasingly difficult to evaluate what standards high-quality online shopping platforms should have. In this conceptual analysis, we discussed how service marketing is working in online shopping platforms and in-depth discuss how important role service has played in the marketing of online shopping platforms. In addition, in the review of relevant literatures, we created a logic comparison among the different opinions, through discussion of the findings of current studies.

## Author Contributions

All authors listed have made a substantial, direct and intellectual contribution to the work, and approved it for publication.

## Funding

This work was supported by Social Science Fund of Jiangsu Province, China (Project No. 18GLB007).

## Conflict of Interest

The authors declare that the research was conducted in the absence of any commercial or financial relationships that could be construed as a potential conflict of interest.

## Publisher's Note

All claims expressed in this article are solely those of the authors and do not necessarily represent those of their affiliated organizations, or those of the publisher, the editors and the reviewers. Any product that may be evaluated in this article, or claim that may be made by its manufacturer, is not guaranteed or endorsed by the publisher.
