# Corrigendum: Service Marketing in Online Shopping Platform: Psychological and Behavioral Dimensions

**DOI:** 10.3389/fpsyg.2021.816413

**Published:** 2021-12-01

**Authors:** Yong Wang, Manci Qi, Liz Parsons, Fu-Sheng Tsai

**Affiliations:** ^1^Business School, Huaiyin Institute of Technology, Huai'an, China; ^2^Doctoral Program, I-Shou University, Taiwan, China; ^3^Management School, University of Liverpool, Liverpool, United Kingdom; ^4^North China University of Water Resources and Electric Power, Zhengzhou, China; ^5^Department of Business Administration, Cheng Shiu University, Kaohsiung, Taiwan; ^6^Center for Environmental Toxin and Emerging-Contaminant Research, Cheng Shiu University, Kaohsiung, Taiwan; ^7^Super Micro Mass Research and Technology Center, Cheng Shiu University, Kaohsiung, Taiwan

**Keywords:** service marketing, online shopping, platform, psychological antecedents, behavioral antecedents

In the published article, there was an error in the author list. An author name was incorrectly spelled as **Manzu Qi**. The correct spelling is **Manci Qi**.

The authors apologize for this error and state that this does not change the scientific conclusions of the article in any way. The original article has been updated.

## Publisher's Note

All claims expressed in this article are solely those of the authors and do not necessarily represent those of their affiliated organizations, or those of the publisher, the editors and the reviewers. Any product that may be evaluated in this article, or claim that may be made by its manufacturer, is not guaranteed or endorsed by the publisher.

